# Supplementary material for: An evolutionary model of rhythmic accelerando in animal vocal signalling
Source: PLoS Comput Biol. 2025 Apr 23;21(4):e1013011. doi: 10.1371/journal.pcbi.1013011 (PMC12054874; doi:10.1371/journal.pcbi.1013011)
Supplement: S1 Appendix — Fig A. Relative approximation error estimates of payoff values. Method A. Maximum acceleration rate Method B. Calculation of expected relative overlap and expected payoff. Method C. Evolutionary process and stationary distribution. Method D. Evolutionary selection and mutation in numerical simulations. (PDF) [file pcbi.1013011.s001.pdf]

An evolutionary model of rhythmic accelerando in animal vocal signalling: S1 Appendix

Yannick Jadoul[1,2,3], Taylor A. Hersh[2,4], Elias Fernández Domingos[3,5], Marco Gamba[6], Livio Favaro[6], Andrea Ravignani[1,2,7,8*]*

[1] *Department of Human Neurosciences, Sapienza University of Rome, Rome, Italy*
[2] *Comparative Bioacoustics Group, Max Planck Institute for Psycholinguistics, Nijmegen, the Netherlands*
[3] *Artificial Intelligence Lab, Vrije Universiteit Brussel, Brussels, Belgium*
[4] *Marine Mammal Institute, Oregon State University, Newport, Oregon, USA*
[5] *Machine Learning Group, Université Libre de Bruxelles, Brussels, Belgium*
[6] *Department of Life Sciences and Systems Biology, University of Turin, Turin, Italy*
[7] *Center for Music in the Brain, Department of Clinical Medicine, Aarhus University, Aarhus, Denmark*
[8] *Research Center of Neuroscience "CRiN-Daniel Bovet", Sapienza University of Rome, Rome, Italy*

*Yannick.Jadoul@uniroma1.it, andrea.ravignani@uniroma1.it

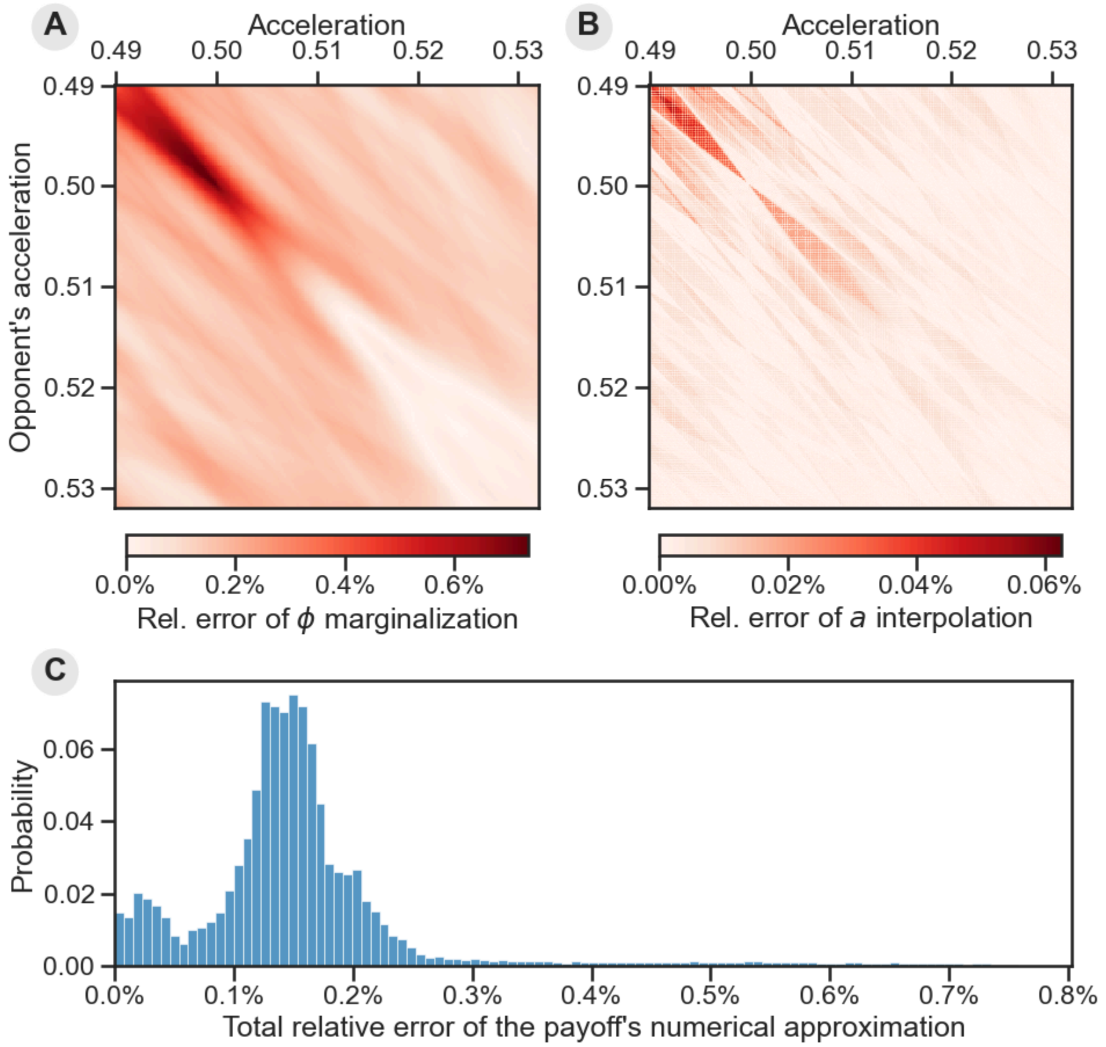

Figure A. *Relative approximation error estimates of payoff values.* ***(A)*** *The payoff's maximum relative error introduced by marginalising over* $\phi \in [-2\pi, 2\pi]$ *is less than 1%, when approximating* $\Pi_{ab} \approx \hat{\Pi}_{ab} = \frac{1}{201} \sum_{i=-100}^{100} O_{a,b,\frac{i}{100}2\pi} - c_a$ *(i.e.,* $N_\phi = 201$*; see "Model of accelerating sequences and overlap" section in methods). The median relative error is less than 0.15%. Relative error was calculated as* $\left| \frac{\Pi_{ab} - \hat{\Pi}_{ab}}{\Pi_{ab}} \right|$*, where* Π_{ab} *was more precisely calculated with* $N_\phi = 2001$*.* ***(B)*** *The payoff's maximum relative error introduced by bivariate spline interpolation of a 100x100 equally-spaced grid is less than 0.1%. The median relative error is less than 0.002%. Relative error was calculated in comparison to an 800x800 grid.* ***(C)*** *The distribution of the total relative error (i.e., including both the marginalisation and interpolation approximation) of* 10^5 *uniformly randomly sampled points in* $[0.49, a_{max}] \times [0.49, a_{max}]$ *shows our numerical approximations are typically within 0.25% of the actual value, validating our conclusions.*

Method A. Maximum acceleration rate

Given a sequence with constant acceleration $a = \frac{IOI_i}{IOI_i + IOI_{i+1}}$, the duration of each IOI is reduced by a constant fraction, such that $IOI_i = r\, IOI_{i-1}$ where $r = \frac{1-a}{a}$. As all durations are scaled wrt. $IOI_1 = 1$, the sequence's final $IOI_{n-1} = r^{n-2} = \left(\frac{1-a}{a}\right)^{n-2}$. Given a syllable duration d that cannot exceed the final IOI duration IOI_{n-1}, the maximum possible acceleration of a modelled sequence is $a_{max} = \frac{1}{1+\sqrt[n-2]{d}}$.

Method B. Calculation of expected relative overlap and expected payoff

Given two accelerating sequences of syllables, with accelerations a and b and a phase offset ϕ between the two, the relative overlap $O_{a,b,\phi}$ can be calculated as the total duration of the intersection of both sequences' syllables divided by the total duration of syllables in one sequence (see insets in Figure 1). As $IOI_1 = 1$, a phase offset ϕ will shift the onset of the second sequence to $\frac{\phi}{2\pi}$. For example, two equally accelerating sequences starting simultaneously (i.e., $\phi = 0$) will have a relative overlap of 1. Two isochronous sequences (i.e., $a = 0.5$) offset by $\phi = \frac{\pi}{2}$ will have a relative overlap of $\frac{10\cdot(0.36 - 0.25)}{10\cdot 0.36} \approx 0.306$. We calculated the exact relative overlap computationally for more complex combinations of sequences. Note that given the constant values for n and d, this relative overlap is symmetric for two sequences with a given phase offset.

Under the assumption that two individuals do not have precise control over the relative phase of their sequences, we marginalised the phase $\phi \in [-2\pi, 2\pi]$ to calculate the expected relative overlap $O_{a,b}$ between two acceleration rates. The values in our analyses are approximated by taking the average relative overlap for $N_\phi = 201$ phase values, equally spaced from -2π to 2π (i.e., $\phi = \frac{i}{100} 2\pi$ with $i \in [-100..100]$; see Figure S1 for further analysis of this approximation's accuracy). As such, the model now provides an expected relative overlap between any pair of acceleration values.

To select a suitable cost function c_a for each possible acceleration a, we took the percentage of the total duration of syllables relative to the sequence's duration and made three additional assumptions. First, we assumed the duration of a syllable compared to the starting IOI is "comfortable" and does not have any cost associated (i.e., the cost of an isochronous or any decelerating sequence is 0). Second, an uninterrupted vocal display of immediately consecutive syllables has cost 1. Third, since a higher percentage of syllable duration during a song means more time spent vocalising and less time to recover between syllables, the relationship between the relative duration of syllables and the cost is modelled as quadratic. Thus, given a percentage of syllable duration p, the only cost function satisfying all three assumptions is $c_a = \left(\frac{p-d}{1-d}\right)^2$ (for $p \geq d$; and $c_a = 0$ for $p < d$). Given the previously described sequence structure, we can calculate $p = \frac{(n-1)d}{\sum_{i=0}^{n-2}\left(\frac{1-a}{a}\right)^i}$.

As outlined in the main text, this cost is subtracted from the expected relative overlap to achieve a total expected payoff $\Pi_{ab} = O_{a,b} - c_a$ for a modelled sequence with acceleration a

with respect to another acceleration rate b. Introducing a cost that depends on the acceleration rate makes the payoff between two sequences non-symmetric.

Method C. Evolutionary process and stationary distribution

The modelled evolutionary process acts over a population of Z individuals, which may adopt any possible acceleration strategies. We represent evolution mathematically as a birth-death process combined with the pairwise comparison rule (Sigmund, 2010; Traulsen et al., 2006). Here, at each time step a random individual i with acceleration a is selected from the population and will undergo mutation with a probability μ. With probability $1 - \mu$ she will have the opportunity to copy the strategy of another randomly selected individual j with acceleration b. If the fitness of j is higher than that of i, i with acceleration a will adopt j's acceleration with probability $q = \left[1 + e^{\beta(f_a - f_b)}\right]^{-1}$, where f_a is the fitness of acceleration strategy a and β represents the intensity of selection. In a population of where each individual i has its own acceleration a_i, the fitness of individual i is $f_i = \frac{1}{Z-1} \sum_{\forall j \neq i} \Pi_{a_i a_j}$; i.e., each individual's fitness equals its expected payoff when uniformly randomly interacting with everyone else in the population.

This adaptive process can be described as a Markov chain. Nevertheless, the complete characterization of this process is almost computationally intractable due to the number of existing states, given the population size and number of strategies (s) we consider. For this reason, we assume the limit of rare mutations $\mu \to 0$ for our analytical calculations, which simplifies the number of states of our system to the number of possible accelerations (Imhof et al., 2005; Traulsen et al., 2006). In this case, we can construct the transition matrix as a function of the fixation probability of a mutant acceleration strategy a in a population in which all individuals adopt the acceleration strategy b, $\rho_{ab} = \left(1 + \sum_{m=1}^{Z-1} \prod_{k=1}^{m} \frac{T^-(k)}{T^+(k)}\right)^{-1}$, where $T^-(k)$ and $T^+(k)$ are the probabilities to, respectively, decrease and increase the number of individuals adopting acceleration a from k to $k - 1$ ($k + 1$). This probability can be calculated as $T^{\pm}(k) = \frac{k}{Z} \frac{Z-k}{Z-1} \left[1 + e^{\mp\beta(f_a - f_b)}\right]^{-1}$. The transition matrix Λ is constructed so that each $\Lambda_{ab} = \frac{\rho_{ab}}{s-1}$ when $a \neq b$ and $\Lambda_{aa} = 1 - \frac{1}{s-1} \sum_{b \neq a} \rho_{ab}$. Finally, the normalised left eigenvector associated with the eigenvalue 1 of Λ gives us the stationary distribution of the Markov chain, which we use to determine the relative prevalence of each strategy in the population.

Method D. Evolutionary selection and mutation in numerical simulations

The evolutionary simulations start out from the same model, interactions, and fitness as presented in Method S3 above. Next, instead of analytically solving the system under the small mutation assumption, we simulated the evolution of the population as follows. As explained in Method S3, the fitness of an individual i with acceleration a_i is $f_i = \frac{1}{Z-1} \sum_{\forall j \neq i} \Pi_{a_i a_j}$. Starting with a population of Z agents that produce isochronous sequences, each subsequent generation of Z agents will have an acceleration sampled with a probability directly proportional to $q \sim e^{\beta f_i}$, representing the intensity of selection (i.e., the weight fitness

has in the selection process). This means the population is updated synchronously after each generation, with offspring produced proportional to q. On top of the inherited acceleration, all agents in the next generation have their acceleration changed by a small, random mutation ($\sim N(0, \sigma)$). This means that the likelihood of significant mutations occurring is proportional to σ.

References

Imhof, L. A., Fudenberg, D., & Nowak, M. A. (2005). Evolutionary cycles of cooperation and defection. *Proceedings of the National Academy of Sciences*, *102*(31), 10797–10800. https://doi.org/10.1073/pnas.0502589102

Sigmund, K. (2010). The Calculus of Selfishness. In *The Calculus of Selfishness*. Princeton University Press. https://doi.org/10.1515/9781400832255

Traulsen, A., Nowak, M. A., & Pacheco, J. M. (2006). Stochastic dynamics of invasion and fixation. *Physical Review E*, *74*(1), 011909. https://doi.org/10.1103/PhysRevE.74.011909
