# Supplementary material for: An evolutionary model of rhythmic accelerando in animal vocal signalling
Source: PLoS Comput Biol. 2025 Apr 23;21(4):e1013011. doi: 10.1371/journal.pcbi.1013011 (PMC12054874; doi:10.1371/journal.pcbi.1013011)
Supplement: S1 Code — (ZIP) [file pcbi.1013011.s002.zip › Models and simulations.html]

Models and simulations


In [1]:

```
import egttools as egt
import numpy as np
import pandas as pd
import scipy.interpolate

import matplotlib.pyplot as plt
import seaborn as sns

import joblib

from tqdm.notebook import trange, tqdm

import collections
import itertools
```

In [2]:

```
%matplotlib inline
```

In [3]:

```
sns.set(style='white')

plt.rcParams['figure.dpi'] = 100
plt.rcParams['figure.figsize'] = (8, 6)
```

In [4]:

```
mem = joblib.Memory("__joblib_cache__", verbose=False)
```

# Model of accelerating sequences and overlap¶

In [5]:

```
from sequence_overlap_model import *
```

In [6]:

```
DURATION = 0.36
N = 10
```

In [7]:

```
@mem.cache()
def get_overlap_and_payoff_matrix():
    acceleration, overlap_matrix = calculate_overlap_matrix(0.49, 200, 201, DURATION, N)
    payoff_matrix = calculate_payoff_matrix(overlap_matrix, acceleration, DURATION, N)
    return acceleration, overlap_matrix, payoff_matrix

acceleration, overlap_matrix, payoff_matrix = get_overlap_and_payoff_matrix()
np.savez("overlap_matrix_0.36_10.npz", acceleration, overlap_matrix)
np.savez("payoff_matrix_0.36_10.npz", acceleration, payoff_matrix)
```

In [8]:

```
plt.figure()
plt.pcolor(acceleration, acceleration, payoff_matrix.T, shading='auto', cmap='viridis')
plt.colorbar()
plt.axvline(0.5, c='k', ls='--')
plt.axhline(0.5, c='k', ls='--')
plt.gca().set_aspect('equal')
plt.show()
```

In [9]:

```
plt.figure()
plt.pcolor(acceleration, acceleration, (payoff_matrix > np.diag(payoff_matrix)[None,:]).T, cmap='binary')
plt.axvline(0.5, c='b', ls='--')
plt.axhline(0.5, c='b', ls='--')
plt.plot([acceleration[0], acceleration[-1]], [acceleration[0], acceleration[-1]], c='r', lw=1)
plt.gca().set_aspect('equal')
plt.show()
```

In [10]:

```
def get_example_sequences(acceleration_a, acceleration_b, interval=(-np.inf, np.inf), plot=True):
    phi = np.linspace(-2 * np.pi, 2 * np.pi, 10001)
    sequence_a = create_sequence(1.0, N, acceleration_a, DURATION)
    sequence_b = create_sequence(1.0, N, acceleration_b, DURATION)
    overlap = np.array([calculate_overlap(sequence_a, sequence_b, p, DURATION) for p in phi])

    selected_phi_interval = (phi >= interval[0]) & (phi <= interval[1])
    selected_phi = phi[selected_phi_interval][np.argmin(np.abs(overlap[selected_phi_interval] - np.mean(overlap)))]

    if plot:
        plt.figure()
        plt.plot(phi, overlap)
        plt.axhline(np.mean(overlap))
        plt.axvline(selected_phi)
        plt.show()
    
    return create_sequence(1.0, N, acceleration_a, DURATION), create_sequence(1.0, N, acceleration_b, DURATION) + selected_phi / (2 * np.pi)

np.savez("example_sequences1.npz", 0.529, 0.527, *get_example_sequences(0.529, 0.527, (0, 2), False))
np.savez("example_sequences2.npz", 0.509, 0.516, *get_example_sequences(0.509, 0.516, (-4, 0), False))
np.savez("example_sequences3.npz", 0.511, 0.501, *get_example_sequences(0.511, 0.501, (4, 7), False))
```

In [11]:

```
@mem.cache()
def get_overlap_and_payoff_matrix_n_phi():
    acceleration, overlap_matrix = calculate_overlap_matrix(0.49, 200, 2001, DURATION, N)
    payoff_matrix = calculate_payoff_matrix(overlap_matrix, acceleration, DURATION, N)
    return acceleration, overlap_matrix, payoff_matrix
```

In [12]:

```
acceleration_n_phi, _, payoff_matrix_n_phi = get_overlap_and_payoff_matrix_n_phi()
np.savez("payoff_matrix_0.36_10_n_phi.npz", acceleration_n_phi, payoff_matrix_n_phi)
```

In [13]:

```
rel_error_n_phi = np.abs(payoff_matrix_n_phi - payoff_matrix) / payoff_matrix_n_phi
np.savez("payoff_rel_error_n_phi.npz", acceleration_n_phi, rel_error_n_phi)
```

In [14]:

```
plt.figure()
plt.pcolor(acceleration, acceleration, rel_error_n_phi, cmap="Reds", vmin=0)
plt.colorbar()
plt.show()

np.max(rel_error_n_phi), np.median(rel_error_n_phi)
```

Out[14]:

```
(0.007339886893303675, 0.001448701416868631)
```

In [15]:

```
@mem.cache()
def get_overlap_and_payoff_matrix_n_acceleration():
    acceleration, overlap_matrix = calculate_overlap_matrix(0.49, 800, 201, DURATION, N)
    payoff_matrix = calculate_payoff_matrix(overlap_matrix, acceleration, DURATION, N)
    return acceleration, overlap_matrix, payoff_matrix
```

In [16]:

```
acceleration_n_acceleration, _, payoff_matrix_n_acceleration = get_overlap_and_payoff_matrix_n_acceleration()
np.savez("payoff_matrix_0.36_10_n_acceleration.npz", acceleration_n_acceleration, payoff_matrix_n_acceleration)
```

In [17]:

```
payoff_matrix_interpolation = scipy.interpolate.RectBivariateSpline(acceleration, acceleration, payoff_matrix, kx=1, ky=1)
payoff_matrix_interpolated = payoff_matrix_interpolation(acceleration_n_acceleration, acceleration_n_acceleration)

rel_error_n_acceleration = np.abs(payoff_matrix_n_acceleration - payoff_matrix_interpolated) / payoff_matrix_n_acceleration
np.savez("payoff_rel_error_n_acceleration.npz", acceleration_n_acceleration, rel_error_n_acceleration)
```

In [18]:

```
plt.figure()
plt.pcolor(acceleration_n_acceleration, acceleration_n_acceleration, rel_error_n_acceleration, cmap="Reds", vmin=0)
plt.colorbar()
plt.show()

np.max(rel_error_n_acceleration), np.median(rel_error_n_acceleration)
```

Out[18]:

```
(0.0006265357353959851, 1.9121771956558195e-05)
```

In [19]:

```
payoff_matrix_interpolation = scipy.interpolate.RectBivariateSpline(acceleration, acceleration, payoff_matrix, kx=1, ky=1)

min_acceleration, max_acceleration = acceleration[0], acceleration[-1]
phi = np.linspace(-2 * np.pi, 2 * np.pi, 2001)

rel_errors = []
rng = np.random.default_rng(42)
for _ in trange(10**5):
    a, b = rng.uniform(min_acceleration, max_acceleration, 2)
    
    sequence_a = create_sequence(1.0, N, a, DURATION)
    sequence_b = create_sequence(1.0, N, b, DURATION)
    overlap = np.mean([calculate_overlap(sequence_a, sequence_b, p, DURATION) for p in phi])
    payoff = calculate_payoff_matrix(np.array([[overlap]]), np.array([a]), DURATION, N)[0, 0]
    
    payoff_interpolated = payoff_matrix_interpolation(a, b)[0, 0]

    rel_errors.append(np.abs(payoff - payoff_interpolated) / payoff)

np.savez("uniformly_sampled_rel_errors.npz", np.array(rel_errors))
```

```
  0%|          | 0/100000 [00:00<?, ?it/s]
```

In [20]:

```
plt.figure()
sns.histplot(x=rel_errors, bins=100, stat='probability')
plt.show()
```

# Evolutionary game-theoretical model¶

In [21]:

```
min_acceleration, max_acceleration = acceleration[0], acceleration[-1]
strategies = np.arange(min_acceleration, max_acceleration, 0.002)
strategies_i = np.argmin(np.abs(acceleration[:,None] - strategies[None,:]), axis=0)

np.max(np.abs(strategies - acceleration[strategies_i]))
```

Out[21]:

```
0.00010468750849124131
```

In [22]:

```
Z = 100
beta = 1
evolver = egt.analytical.StochDynamics(len(strategies), payoff_matrix[np.ix_(strategies_i, strategies_i)], Z)
```

In [23]:

```
plt.figure()
sns.heatmap(payoff_matrix[np.ix_(strategies_i, strategies_i)].T, cmap='viridis', annot=False, linewidths=.5)
plt.gca().set_xticklabels([f'{s:.3f}' for s in strategies], rotation=90)
plt.gca().set_yticklabels([f'{s:.3f}' for s in strategies], rotation=0)
plt.show()
```

In [24]:

```
transition_matrix, fixation_probabilities = evolver.transition_and_fixation_matrix(beta)
stationary_distribution = egt.utils.calculate_stationary_distribution(transition_matrix)
np.savez("transition_matrix_and_probabilities.npz", transition_matrix=transition_matrix, fixation_probabilities=fixation_probabilities)
```

In [25]:

```
fig, ax = plt.subplots(figsize=(6, 6), dpi=150)
G = egt.plotting.draw_stationary_distribution([f'{s:.3f}' for s in strategies], 1/Z, fixation_probabilities, stationary_distribution, node_size=500, font_size_node_labels=6, font_size_edge_labels=4, font_size_sd_labels=7, max_displayed_label_letters=10, edge_width=1, min_strategy_frequency=0.00, ax=ax)
plt.axis('off')
plt.show()
```

# Numerical simulations¶

In [26]:

```
def simulate_evolution(payoff, min_acceleration, max_acceleration, n_agents, mutation_sigma, selection_strength, n_iterations, rng=None):
    if rng is None:
        rng = np.random.default_rng()
    
    agents = 0.5 * np.ones(n_agents)
    accelerations = np.zeros((n_iterations + 1, n_agents))
    accelerations[0,:] = agents

    for i in trange(n_iterations):
        agents.sort()
        pairwise_payoffs = payoff(agents, agents)
        np.fill_diagonal(pairwise_payoffs, np.nan)
        average_payoffs = np.nanmean(pairwise_payoffs, axis=1)
        relative_fitness = np.exp(selection_strength * average_payoffs)
        relative_fitness /= relative_fitness.sum()
        agents = rng.choice(agents, size=len(agents), p=relative_fitness) + rng.normal(loc=0, scale=mutation_sigma, size=len(agents))
        agents = np.maximum(min_acceleration, np.minimum(max_acceleration, agents))
        accelerations[i+1,:] = agents

    return accelerations
```

In [ ]:

```
payoff_interpolation = scipy.interpolate.RectBivariateSpline(acceleration, acceleration, payoff_matrix, kx=1, ky=1)
min_acceleration, max_acceleration = acceleration[0], acceleration[-1]

N_AGENTS = [50, 100, 200]
MUTATION_SIGMA = [5e-4, 1e-4, 5e-5, 1e-5]
SELECTION_STRENGTH = [1, 10]  # 0.1,
REPEATS = 5
GENERATIONS = 10**5

combination_results = np.empty((len(N_AGENTS), len(MUTATION_SIGMA), len(SELECTION_STRENGTH), REPEATS, GENERATIONS + 1, 3), dtype='float64')

for n_agents, mutation_sigma, selection_strength, i in tqdm(list(itertools.product(N_AGENTS, MUTATION_SIGMA, SELECTION_STRENGTH, range(REPEATS)))):
    rng = np.random.default_rng(42 + i)
    accelerations = simulate_evolution(payoff_interpolation, min_acceleration, max_acceleration, n_agents, mutation_sigma, selection_strength, GENERATIONS, rng)
    
    n, m, s = N_AGENTS.index(n_agents), MUTATION_SIGMA.index(mutation_sigma), SELECTION_STRENGTH.index(selection_strength)
    
    combination_results[n, m, s, i, :, 0] = np.median(accelerations, axis=1)
    combination_results[n, m, s, i, :, 1] = np.quantile(accelerations, 0.25, axis=1)
    combination_results[n, m, s, i, :, 2] = np.quantile(accelerations, 0.75, axis=1)

np.savez("simulations_combinations_results.npz", combination_results, N_AGENTS, MUTATION_SIGMA, SELECTION_STRENGTH)
```

In [ ]:

```
N_AGENTS, MUTATION_SIGMA, FITNESS_FACTOR = 100, 1e-04, 1

rng = np.random.default_rng(42)

START = 0
for i in trange(100):
    if i < START:
        rng = np.load(f"simulations/simulations_rng_100_0.0001_1__{i+1}.npy", allow_pickle=True)[()]
        continue

    np.save(f"simulations/simulations_rng_100_0.0001_1__{i}.npy", rng)

    accelerations = simulate_evolution(payoff_interpolation, min_acceleration, max_acceleration, N_AGENTS, MUTATION_SIGMA, FITNESS_FACTOR, 10**6, rng)

    np.save(f"simulations/median_accelerations_100_0.0001_1__{i}.npy", np.median(accelerations, axis=1))
    np.save(f"simulations/last_generations_accelerations_100_0.0001_1__{i}.npy", accelerations[-100:,:])
```
