# Supplementary material for: An evolutionary model of rhythmic accelerando in animal vocal signalling
Source: PLoS Comput Biol. 2025 Apr 23;21(4):e1013011. doi: 10.1371/journal.pcbi.1013011 (PMC12054874; doi:10.1371/journal.pcbi.1013011)
Supplement: Attachment — Submitted filename: Revision 1 - Rebuttal letter.pdf [file pcbi.1013011.s004.pdf]

***Reviewer #1***: *This manuscript is about an EGT model of the dynamics of accelerando as an evolving trait in a population of vocalizing animals. In the model, fitness (i.e., payoff) is operationalized by the net overlap of the sequences of two interacting individuals. There is a trade-off between acceleration and production effort, which eventually leads to the existence of an evolutionarily stable strategy representing a stable amount of accelerando in the population (actually, in 'evolutionary invasion analysis' terminology it's a convergent stable strategy, McGill et al. 2007).*

*I know relatively little about animal communication, so I cannot say much about the relevance and soundness of this study in this regard. For what it's worth, the African penguin example looks good to me. However, as far as the presented modeling work is concerned, I found this to be an excellent manuscript. The model is reasonably elegant, clearly described, as are its results, and the discussion is very insightful (just a few remarks, see below). The supplementary document is very informative (in fact, some of the information could be moved to the main manuscript). Overall, I have little to complain about, but I do have a couple of suggestions and/or questions that the authors might want to consider.*

**We thank the reviewer for this succinct summary of the manuscript, for the kind words on the presented modeling, and the extremely constructive feedback here and below! We are very glad to read the positive assessment of our model and its subsequent theoretical and computational analysis, and we have done our best to resolve the points raised below.**

**Thank you for the reference and terminology as well. We have included it in our manuscript (lines 258–259).**

** Payoff is defined as overlap minus production costs. Crucially, production only affects one individual, which in turn leads to asymmetries in the payoff matrix, as the authors point out. I guess, perceptual costs exist as well (because sequences with very short pauses between syllables might be difficult to decompose). Is there a reason why perceptual costs are not modeled? Or is the argument that perceptual costs are very small compared to articulatory costs?*

**Thank you. This was indeed our line of reasoning, as we assumed the costs associated with the production of the loud songs to be prevailing compared to any other cost. While most costs, including perceptual ones, rely on an always active nervous system, production can make an organism incur additional significant costs. In addition, for the specific case of the African penguins, we are still learning more about perception and whether e.g. segmentation plays an important role, if any at all. So besides having a much lower energetic cost, it is not as straightforward to determine which aspects of perception should be captured in such a modelled cost. That said, we appreciate the question and have added a clarification to the manuscript to make this reasoning more explicit (lines 206–207).**

** Maybe say in the paragraph starting with "Next, given two accelerating sequences…" that*

*phases between the two sequences are determined randomly. It is specified in S2, I know, but this information would have helped at this point in the main manuscript.*

**Thank you for pointing this out. This is a helpful perspective, and we have improved the manuscript as suggested (lines 201–204).**

** Figure 1 is good, but for the purpose of this manuscript, the pairwise invasibility plot (PIP) computed in the notebook "Models and simulations" (i.e., the black & white plot created in "In [9]") is much more informative than the continuous payoff matrix. This is because from the PIP one can immediately infer that there is an attracting ESS (i.e., a continuously stable strategy) in the middle of the trait space. And one can see that the isochrony strategy does not even represent an evolutionary equilibrium. I strongly recommend putting this plot next to the red-yellow one in Fig 1 since there is some space left anyway.*

**We thank the reviewer for this very constructive suggestion. We initially feared that, to the general reader, this might be a somewhat too technical graph and therefore did not add it to the main manuscript. But we are happy to hear the reviewer believes this to be a useful plot and have combined it into Figure 1! We have updated the caption accordingly, and have done our best to ensure the new figure is as accessible to the general readership as possible.**

** Another thought on the PIP if you think of it in terms of evolutionary invasion analysis (i.e., assuming that mutations are so rare that new mutants always face a homogeneous resident population – Brännström et al. 2013): Interestingly, it displays two islands (one in the lower left corner, above the diagonal, and one in the upper right quadrant below the diagonal). I understand that the authors are interested in small mutational steps of accelerando, but as a matter of fact, this payoff matrix allows for rock-paper-scissor dynamics due to these islands if larger jumps are allowed. That is, theoretically, the evolutionary dynamics could get stuck in a loop of alternating high-level accelerando strategies without approaching the ESS in the middle. See Gyllenberg and Service (2011) for more details. I find this is actually an interesting scenario, but I leave it for the authors to decide in how far they want to comment on this.*

**This is an interesting observation; thank you for mentioning it. We have been in doubt on whether to include this in the main text, but in the end decided against it for two main reasons. Primarily, we want to avoid adding more complexity to the manuscript, as to keep it as natural and accessible as possible for the readers. Additionally, we have added a heatmap of the difference in payoff to Figure 2A, where the positive regions correspond to the possible invasive strategies (i.e., the black region in Figure 1, as suggested in the reviewer's previous comment). This plot shows that the two islands mentioned have a relatively low difference in payoff, especially compared to the other regions. We believe this explains why these evolutionary dynamics do not feature strongly in the subsequent analyses, even though our analysis of the 21 discrete acceleration rates (under the small mutation assumption) did not impose any limitations on the size of the jumps. Thus, we hope that the reviewer agrees that**

**including this nevertheless interesting finding is probably not worth the distraction from the main explanation of the model and its results.**

** Discussion section, "If overlap benefits individuals, accelerating sequences are a controlled way of maximising vocal overlap among competing signalers.": I think a crucial point also is that this holds true, on average, *even if there are phase differences* (i.e., if both do not start signaling at the same time). I can imagine that isochrony is a risky strategy if there is lack of control about phases because if you're not lucky you end up having zero overlap (on the other hand, if you *are* lucky, you might have 100% overlap, so on average it might balance out). This brings me to the following question: how central is the assumption of having no control over the relative phase for the model/analysis? Could accelerando be viewed as a strategy to compensate for the lack of control over phases?*

**We thank the reviewer very much for this comment. We completely agree that this is a correct and intuitive way of summarizing and interpreting the results regarding phase offset and overlap. If the reviewer allows us, we would like to include this interpretation in our Discussion and have done so (lines 349–356).**

** Paragraph starting with "Crucially, our simulated individuals are only allowed" in the discussion: I am not sure if I can buy the argument in this paragraph. At the surface, it looks like a 'reductio ad absurdum' line of argumentation, but it doesn't seem to be entirely logical to me. E.g., "One may argue that a faster, purely isochronous signal could perform equally well. But if [a higher?] duty cycle could explain everything, we would find equilibria for highest levels of acceleration, rather than the intermediate acceleration we do find." Maybe I have misunderstood it, but isn't the whole point that the mid-level ESS emerges because of the trade-off with production costs? Obviously, fast isochronous signals should also have higher costs than not so fast ones. Similarly, "this hypothesis is difficult to test because it would practically entail comparing an infinite number of individual rhythmic patterns." – not a convincing point in the present context since you *are* comparing an infinite number of different strategies (the accelerando trait is continuous). Finally, "Following this line of thought, there may not even be a need for rhythm, as an uninterrupted long sound could grant maximum overlap." I mean, yes, if overlap was the only factor. But an uninterrupted long sound violates so many other fundamental assumptions (e.g., that we are talking about syllable sequences here) that I am not sure if this is a valid (valid in the sense of 'legit absurd') conclusion. Overall, the paragraph left me a bit puzzled.*

**We thank the reviewer for their critical reading of the manuscript. We are glad that this is the only point where the reviewer finds holes in our logical reasoning, but we concede that the reasoning is too unclear and somewhat convoluted. This paragraph was meant to pre-empt doubts regarding the relevance of our assumptions and model, but based on the reviewers' comments, this might have been somewhat premature. As such, we have removed this argument from the Discussion and replaced it with the answer to the reviewer's previous comment (lines 346–356), as well as with the additional paragraph discussing the complex feedback loop typical of**

**evolutionary models we wrote in response to reviewer 2 (lines 357–372). Again, we thank the reviewer for the fair and constructive feedback.**

** Article missing in “We include such [a] selective pressure …” on page 5 of the manuscript.*

**Thank you for pointing this out. We have fixed it accordingly.**

** Supplement, S3: How is fitness f_a defined in S3? (It is only defined for S4 below.)*

**We thank the reviewer very much for this thorough reading, and even pointing out this oversight in the supplement! Something must have gone wrong indeed, when editing and reshuffling the supplement draft. The fitness calculation is the same as in S4, but was indeed mentioned too late. We have now fixed this.**

*References:*

*Brännström, Å., Johansson, J., & Von Festenberg, N. (2013). The hitchhiker’s guide to adaptive dynamics. Games, 4(3), 304-328.*

*Gyllenberg, M., & Service, R. (2011). Necessary and sufficient conditions for the existence of an optimisation principle in evolution. Journal of mathematical biology, 62, 359-369.*

*McGill, B. J., & Brown, J. S. (2007). Evolutionary game theory and adaptive dynamics of continuous traits. Annu. Rev. Ecol. Evol. Syst., 38(1), 403-435.*

***Reviewer #2***: *The MS is on the evolution of overlapping calls. It is an interesting topic; in many well-documented studies, signalling animals actively avoid call overlap, so why have some species (like the African penguin) produce calls with an accelerated call rate with and increase of overlapping calls?*
*The authors have made an evolutionary model of call evolution where the fitness is calculated from call overlap minus a cost (the physiological cost of call production, increasing for accelerated call rates). This model produces an increase in accelerated call rates and call overlap in the population.*

**We thank the reviewer for the succinct, correct summary of our manuscript and model. We appreciate the constructive comments below and have done our best to answer and resolve them.**

*As far as I see, this model is completely circular. The selection pressure benefits overlap, and accelerated calls will increase overlap(unless overlap is actively avoided), so evidently, if natural selection works as an algorithm, ( which it does), it will produce the result following from the initial hypothesis.*

**Thank you for this comment. We agree with most of the points you raise, but believe that there are two crucial aspects of our study to point out, which we hope convince the reviewer that our model is indeed *not* circular nor its results trivial.**

**1) A crucial point of evolution and evolutionary models is that the evolving strategies/traits of individuals in a population directly impact the fitness landscape. While certain evolutionary adaptations will have a clear benefit, other evolutionary advantages are dependent on the rest of the population. The model presented here is such a case, as the fitness of a certain acceleration strategy directly depends on the proportion of other strategies present in the population. In Figure 1, the maximum payoff for an individual is achieved with an acceleration of around 0.525 *if* the rest of the population accelerates at rates around 0.53. However, this is not the acceleration rate we see at the model and simulation's equilibrium (Figures 2 and 3). The reason for this is that, at high acceleration rates, the increased fitness of a *slightly* lower rate than the rest of the population would over time result in more individuals accelerating at a lower rate. As the whole population's acceleration rate changes over time, so does the evolutionary landscape and maximum payoff which can be achieved by each individual. As such, while our model might seem simple and its results perhaps obvious, they comprise more than the mere tradeoff between overlap and cost faced by each individual. Evolutionary models and computational simulations are indispensable to capture the complex feedback loop between individual strategies and the overall population, even when the results appear trivial.**

**2) Additionally, the resulting overlap between accelerating sequences was not straightforward or expected to us. In hindsight, the increased overlap can be interpreted intuitively: Accelerating rhythms will have a higher duty cycle and therefore have an a priori higher chance of overlap. Our model explicitly quantifies the interaction of this effect and the assumption of random offsets between sequences. As reviewer 1 writes, one way of further understanding this is by realising isochrony**

**is a risky strategy if there is lack of control over the phase offset. While two sequences can end up with close to 100% overlap, an unlucky offset will result in 0% overlap. Based on our results, accelerando could be viewed as a strategy to compensate for the lack of control over phases. Future work should model different levels of control over the phase offset and study how it influences the resulting evolutionary dynamics.**

**Revising our manuscript in the light of this comment however, we do entirely agree with the reviewer that these arguments were not made as explicitly as they should have been, and that we took such interpretation by the reader for granted. As such, we are now including both parts as a paragraph in the Discussion (lines 346–356 and 358–373). We hope the reviewer agrees that these two additional Discussion paragraphs address and clarify the raised concerns.**

*Another problem, I think, is that the behavioral context of the call is not addressed in the MS. In the video in the supplementary materials, we see a lot of vocal interactions of penguins with different call types. The amount of call overlap appears to be rather small in this video, at least for the ecstatic call, normally uttered by single birds (but inducing others to follow in chorus, Favaro et al. 2014) . Also, the ecstatic call is long, and overlap with other calls may simply not be very important for example for localization of the caller. Also, whereas it is clear that animals often avoid call overlap, overlap can be an indication of increased aggression in agonistic encounters. I think these are competing hypotheses - either that the overlap is incidental, and selection pressure neutral, but there could be a selection pressure for increasing duty cycle of the call, or that overlap has a direct agonistic function. In species that actively avoid call overlap, the behavioral context - of mate selection and localization - is different.*

**Thank you for your feedback. We have updated the introduction to make it clear that ecstatic display dongs (EDSs) are primarily produced by male penguins during breeding periods (line 127). EDSs are typically composed of three different types of syllable, which we believe are the “different call types” the reviewer has picked up on (Favaro et al., 2014). As the reviewer points out, overlap with other calls may not be important if the goal of an EDS is for the caller to be localized, but in actuality the exact function of EDSs remains unknown. These vocal displays seem to play a role in nest territory defense, mate recognition, and mate attraction, but how they bolster or facilitate such displays is unknown (Calcari et al. 2021; Favaro et al. 2014; Baciadonna et al. 2022; Favaro et al. 2020). We have also included this latter sentence in the manuscript, as further context (lines 130–133).**

**While the question of the potential function of the African penguins’ songs is indeed fascinating and provided the initial spark of inspiration for the current model, and we hope that our work can contribute to the investigation of the EDSs’ potential function(s), we do not believe our current model can at this point provide a clear solution to this larger biological question. Our paper is, at its core, a modelling paper that uses African penguin vocalizations as an example, and not an African penguin paper per se. As such, we hope that the reviewer agrees with the delicate balance we**

**have tried to strike between, on the one hand, grounding our model in a biologically observed behavior, and on the other hand, not drawing too strong conclusions based on a first, general model of accelerando and overlap.**

*specific comments:*
*Introduction: I think you need to discuss the different behavioral contexts here. The behavioral task of selecting and localizing a potential mate from afar is very different from communicating in a social group and again different from the communication between two agonists. Humans without hearing impairment have no great problems with call overlap (in cocktail party-situations, for example).*

**Thank you for helpfully pointing this potential improvement to our manuscript. We agree we had not made the link explicit enough between the behavioral context and our model. If the reviewer permits, we have now added these potential behavioral context and made clear that our model is agnostic to this specific context: "And so, while we do not intend to explain the evolution of African penguins' vocal behaviour in particular, the known rhythmic characteristics of these specific songs provide inspiration for a general model of accelerating sequences. Likewise, our model is agnostic with respect to the specific function or behavioural task. While in nature, the behavioural context for such vocal displays might be as diverse as electing and localizing a potential mate from afar, communicating in a social group, or agonistically competing with other individuals (Greenfield, 1994; Hartbauer et al., 2014; Herman, 2017; Legett et al., 2019; Ryan, 1992), the model described below can apply any such context where overlap could be hypothetically beneficial to an individual." (lines 150–158).**

*l.14 from end of introduction: 'leading to a dynamic, cacophonous soundscape' - it is not necessarily cacophonous for the penguins that will have cues based on location and distance to the other penguins in the colony.*

**We thank the reviewer for pointing this out. While we did not intend to mean to imply any sort of characteristic of the penguins' perception of these songs, we understand the word "cacophonous" could be overinterpreted. Instead, we have replaced the word with the more neutral description of "noisy", in order to still convey the loud, disperse nature of these songs when produced in a dense colony (line 136).**

*Discussion: ,l. 3 'If overlap benefits individuals...'- I think it is clear that this assumption will produce acceleration of call rate, without having to run an evolutionary model. You need to consider other hypotheses (as outlined above)*

**We thank the reviewer for bringing this perspective to our attention. We understand the previous version of the manuscript did not discuss this concern in enough detail. We would like to refer to our previous, extended answer above, and hope that the**

**additional paragraphs in the Discussion (lines 346–356 and 358–373) help clarify why we believe our results do not immediately follow from the setup of our model.**

Reviewer #3: *This paper sets out to demonstrate that a simple selective pressure to maximize expected overlap in vocalizations of a fixed duration, while minimizing the physiological cost, causes an initially isochronous population — that does not have any mechanism of synchrony — to evolve towards producing increasingly accelerating sequences until a population-wide equilibrium of rhythmic accelerando is reached.*

**We thank the reviewer for the concise summary of the manuscript, and are glad that our main message came across correctly.**

*I found the paper a difficult read initially, but I think I understood by the end. I simply recommend that the authors bring some of the information from the discussion into the introduction to make understanding of the model easier and more intuitive.*

*Caveats: I am a modeler, but I don't work in the area of evolution. I am not very familiar with the animal literature. And I I did not have time to work through the entire model. I think I followed the basic idea, but some pretty simple questions arose for me. Perhaps addressing these questions up front will help the authors communicate better these findings with a general readership.*

**Thank you for raising this point. We very much appreciate the suggestions on how to improve the readability, as we aim for the manuscript to be as accessible as possible, especially to readers with a general modelling background. Based on the summary above and the points below, we are happy that the reviewer has understood the gist of our model and study, despite these raised doubts. We have taken the comments below and those of the other reviewers into account to improve the manuscript's readability, and have paid extra attention to this point in general during revision! We hope these revisions help further clarify some matters to the reviewer and have made the manuscript easier for future audiences to understand.**

*I'm not sure I understand the first model. If each individual has it's own acceleration rate, then how can it be said that acceleration is evolving? It there to begin with. Is it because some rates are = .5? The mechanism for acceleration is assumed to exist and appear at random, and then accelerating individuals are selected for. It that right? This could be made clearer.*

**We thank the reviewer for this question. The first model, the evolutionary game theory analysis under the small mutation assumption, assumes a population where individuals get replaced by new individuals based on their difference in fitness. A full, in-depth explanation of this model and its analysis is available in the Supplementary Material, as we assumed it would reduce the paper's accessibility if we had included it in the main manuscript. However, we agree that it should be briefly mentioned in the manuscript, and have added a clarifying sentence (lines 222–223).**

*Why doesn't synchrony (& isochrony) always result in the highest overlap? Can you answer this with an intuitive explanation. If two animals are changing tempo (in any direction) at different rates, overlap should be less than it is for perfect synchrony, right?*

*If animals don't reposed to (control) relative phase, then how to they maintain any type of synchrony? They just begin together and proceed at what happens to be an identical rate?*

**Thank you for pointing out this potential point of confusion. The reviewer's latter description is exactly what happens in the model, indeed. A key assumption of the model is that individuals have no control over the relative phase. As such, we compute the *expected* amount of overlap that two individuals would have (i.e., on average across multiple interactions). We have tried to make this clearer to the readers (lines 201–204).**

**This latter part also indirectly answers the reviewer's former question. Isochrony could be a perfect overlap between two sequences, but only on the condition that individuals can coordinate or synchronize through fine-tuned control over their relative phase. Reviewer 1 phrased this succinctly as "Could accelerando be viewed as a strategy to compensate for the lack of control over phases?", an insightful point which we now include in the manuscript (lines 349–356).**

*10^6 means 100,000 generations? Is that biologically realistic?*

**Thank you for raising this point. Our answer here is twofold:**

**1) The number is not really meant to be as realistic to this level. Our whole evolutionary model/simulation is of course a gross simplification of the evolutionary process and does not intend to capture the full intricacy of biological evolution. The generations, being completely replaced at every step of the simulation, are one such unrealistic simplification. However, our main goal is to demonstrate the hypothetical link between overlap and accelerando and the existence of a potential evolutionary path.**

**2) Yes, with a quick back-of-the-envelope calculation, we can see that a million generations can be the sort of very rough scale on which evolution takes place. As an example, estimates for the last known ancestor to humans, chimpanzees, and bonobos vary between 5 and 10 million years ago, showing speciation typically happens over millions of years. When combined with a time between generations of a handful of years (African penguins reach sexual maturity from 4-6 years old), $10^6$ generations is not an unrealistic number for our generic model.**

*Could this phenomenon occur if individuals were trying to synchronize, but each had a different rate, and they tried to adapt to one another's rate?*

**We thank the reviewer for this insightful question. In our model presented here, each individual has a single, fixed acceleration rate, which cannot change throughout a display/accelerating sequence. One reason for this is that there is currently little evidence for this type of vocal flexibility in African penguins. The empirical work (Hersh et al., in review) which inspired the current model also shows limited variability in acceleration rates throughout a song. Vice versa, this restriction in the current model and its results show that a more complex mechanism for synchronization is not strictly necessary to evolve the production of acceleration vocal displays.**

**We do agree that the reviewer's idea of investigating the synchronization during displays is a very interesting one. However, this would be such a drastic change that it would fundamentally change the model and require the whole study to be reframed from an entirely different perspective. As such, we hope that the reviewer agrees that this is out of the current manuscript's scope, but we believe it merits inclusion as a future direction and have done so (lines 38–383).**

*As I read this paragraph, it addresses many of my questions in one way or another: "they cannot adjust the note delay with respect to each other. In addition, our simulated individuals cannot change the (initial) tempo of their vocalisation; i.e., they cannot evolve to produce faster isochronous sequences instead of gradually accelerating ones. The choice of these two assumptions had one main underlying reason. Because isochronous tempo and relative phase are the most common parameters in models of animal rhythms, we decided to avoid manipulating those two parameters to test whether rhythmicity could nonetheless evolve."*

*Could some of these things be said sooner than in the discussion. For example, I don't think you said, up to this point, that they begin at a random delay. If that's true, should be said clearly sooner.*

**Thank you very much for raising this point. We have received similar feedback from the other reviewers and have therefore paid extra attention to this.**

**We have added the information regarding a sequence's random offset/delay/phase to the main manuscript (line 201–204); before, this was only mentioned so explicitly in the Discussion and the supplement, and we agree it needs to be more prominent.**

**We have now also very explicitly noted that the first IOI of all sequences is fixed at $IOI_1$ = 1 (lines 167–169). On revision, we completely agree that both these assumptions in the model were not as clear as they should have been.**

**We have opted to keep the discussion and justification of these assumptions in the Discussion section of the manuscript, and hope that the reviewer agrees that this is where these sentences fit best into the overall narrative.**

*I think the statement on the limitations is reasonable and the authors are not claiming anything beyond what their results show.*

**Thank you for the kind assessment of this part of the discussion! It is of course impossible to make a model without any assumptions or simplifications, but we believe that explicitly spelling these out is key to a correct presentation and understanding of the study. As such, we are delighted to learn that the reviewer considers this aspect to be satisfactory in our manuscript.**

*Also, on page 2, line2: "emergency" should be "emergence"*

**Thank you very much for pointing out this typo. We have fixed it accordingly.**

***Journal Requirements:***

*1) Please ensure that the CRediT author contributions listed for every co-author are completed accurately and in full.*
*At this stage, the following Authors/Authors require contributions: Yannick Jadoul, Taylor Hersh, Elias Fernández Domingos, Marco Gamba, Livio Favaro, and Andrea Ravignani. Please ensure that the full contributions of each author are acknowledged in the "Add/Edit/Remove Authors" section of our submission form.*
*The list of CRediT author contributions may be found here:*
*https://journals.plos.org/ploscompbiol/s/authorship#loc-author-contributions*

**Thank you for bringing this missing section to our attention. We have added it now to the manuscript and will enter it into the submission system.**

*2) We ask that a manuscript source file is provided at Revision. Please upload your manuscript file as a .doc, .docx, .rtf or .tex. If you are providing a .tex file, please upload it under the item type u2018LaTeX Source Fileu2019 and leave your .pdf version as the item type u2018Manuscriptu2019.*

**Thank you, we have now done so.**

*3) Please provide an Author Summary. This should appear in your manuscript between the Abstract (if applicable) and the Introduction, and should be 150-200 words long. The aim should be to make your findings accessible to a wide audience that includes both scientists and non-scientists. Sample summaries can be found on our website under Submission Guidelines:*
*https://journals.plos.org/ploscompbiol/s/submission-guidelines#loc-parts-of-a-submission*

**Thank you. We have now added an Author Summary to the main manuscript file.**

*4) Please upload all main figures as separate Figure files in .tif or .eps format. For more information about how to convert and format your figure files please see our guidelines:*
*https://journals.plos.org/ploscompbiol/s/figures*

**Thank you for the reminder. Given that this was still a major revision, several figures still changed. In case our revised manuscript will go out for review again, the figures might not yet be final. As such, we hope the editors and journal are OK if we only upload the final figures on acceptance and final revision.**

*5) We have noticed that you have uploaded Supporting Information files, but you have not included a list of legends. Please add a full list of legends for your Supporting Information files after the references list.*

**Thank you. We have added this to the end of the main manuscript.**

*6) We are unable to open the following Supporting Information file: code.zip. Please kindly revise as necessary and re-upload.*

**Thank you. We have double-checked the supporting information and will make sure we upload a readable version of it.**

*7) Some material included in your submission may be copyrighted. According to PLOSu2019s copyright policy, authors who use figures or other material (e.g., graphics, clipart, maps) from another author or copyright holder must demonstrate or obtain permission to publish this material under the Creative Commons Attribution 4.0 International (CC BY 4.0) License used by PLOS journals. Please closely review the details of PLOSu2019s copyright requirements here: PLOS Licenses and Copyright. If you need to request permissions from a copyright holder, you may use PLOS's Copyright Content Permission form.*
*Please respond directly to this email and provide any known details concerning your material's license terms and permissions required for reuse, even if you have not yet obtained copyright permissions or are unsure of your material's copyright compatibility. Once you have responded and addressed all other outstanding technical requirements, you may resubmit your manuscript within Editorial Manager.*
*Potential Copyright Issues:*

*- Figure 1. Please confirm whether you drew the images / clip-art within the figure panels by hand. If you did not draw the images, please provide a link to the source of the images or icons and their license / terms of use; or written permission from the copyright holder to publish the images or icons under our CC BY 4.0 license. Alternatively, you may replace the images with open source alternatives. See these open source resources you may use to replace images / clip-art:*
*- https://commons.wikimedia.org*
*- https://openclipart.org/.*

**Thank you for pointing this out. The small penguin figures in Figure 1 were in fact drawn by one of our co-authors (Prof. Dr. Marco Gamba), and are available for us to use in the manuscript.**

*8) We note that your Data Availability Statement is currently as follows: "The manuscript contains no data. All scripts/code appear as supplement.". Please confirm at this time whether or not your submission contains all raw data required to replicate the results of your study. Authors must share the “minimal data set” for their submission. PLOS defines the minimal data set to consist of the data required to replicate all study findings reported in the article, as well as related metadata and methods (https://journals.plos.org/plosone/s/data-availability#loc-minimal-data-set-definition).*
*For example, authors should submit the following data:*
*- The values behind the means, standard deviations and other measures reported;*
*- The values used to build graphs;*
*- The points extracted from images for analysis..*
*Authors do not need to submit their entire data set if only a portion of the data was used in the reported study.*
*If your submission does not contain these data, please either upload them as Supporting Information files or deposit them to a stable, public repository and provide us with the relevant URLs, DOIs, or accession numbers. For a list of recommended repositories, please see https://journals.plos.org/plosone/s/recommended-repositories.*
*If there are ethical or legal restrictions on sharing a de-identified data set, please explain them in detail (e.g., data contain potentially sensitive information, data are owned by a third-party organization, etc.) and who has imposed them (e.g., an ethics committee). Please*

*also provide contact information for a data access committee, ethics committee, or other institutional body to which data requests may be sent. If data are owned by a third party, please indicate how others may request data access.*

**This is correct. Our modelling paper does not contain any dataset, except for the one generated by the model itself. All necessary code to do so is uploaded as supplementary data.**
